# Self-efficacy and diabetes prevention in overweight South Asians with pre-diabetes

**DOI:** 10.1136/bmjdrc-2018-000561

**Published:** 2018-10-15

**Authors:** Catherine E Cioffi, Harish Ranjani, Lisa R Staimez, Ranjit Mohan Anjana, Viswanathan Mohan, Mary Beth Weber

**Affiliations:** 1 Nutrition and Health Sciences Doctoral Program, Laney Graduate School, Emory University, Atlanta, USA; 2 Madras Diabetes Research Foundation, Chennai, India; 3 Emory Global Diabetes Research Center, Hubert Department of Global Health, Emory University, Atlanta, USA

**Keywords:** prediabetic phenotype, prevention, self-efficacy, Asian Indians, psychosocial

## Abstract

**Objective:**

We evaluated the effects of a diabetes prevention itervention on self-efficacy (SE) and the associations between SE and diabetes-related outcomes among overweight Asian Indian adults with pre-diabetes in a randomized controlled translational trial (the Diabetes Community Lifestyle Improvement Program, D-CLIP).

**Research design and methods:**

Data were obtained from 550 adults who were randomized to a diabetes prevention program or standard of care. Dietary and exercise-related SEs were measured at baseline, core intervention completion (4  months), and annually until the end of follow-up (3 years or diabetes diagnosis). Mixed-effects regressions described changes in SE over time by treatment group. Among treatment participants, multivariable-adjusted models described associations of SE at baseline and intervention completion with diabetes incidence and other secondary outcomes (weight, waist circumference (WC), exercise, and energy intake).

**Results:**

From baseline to 4  months, dietary (β=10.3, p=0.04) and exercise (β=0.49, p=0.04) SE increased significantly in the treatment arm only; however, this increase from baseline was no longer significant at later time points. Among treatment participants, there was no association of dietary or exercise SE with diabetes incidence, but baseline exercise SE was independently associated with improved weight, WC, and exercise at 4  months (p<0.05). Change in exercise SE from baseline to intervention completion also predicted increased exercise at 4, 12, and 24 months (p<0.05).

**Conclusions:**

Exposure to D-CLIP resulted in improved SE at treatment completion, but this effect was not sustained over longer follow-up. Several short-term and long-term secondary outcomes, but not diabetes risk, were significantly associated with exercise SE, suggesting this psychosocial trait may facilitate success in achieving certain health goals.

**Trial registration number:**

NCT01283308.

**Significance of this study**

**What is already known about this subject?**

- The influence of psychosocial factors on the effectiveness of translational diabetes prevention program has been studied in certain populations, but thus far no studies have examined this in Asian Indians.

**What are the new findings?**

- Among Asian Indian adults who participated in a community-based, translational diabetes prevention program, increased exercise self-efficacy at baseline predicted improved health outcomes at intervention completion, and increased change from baseline to intervention completion predicted increased exercise at follow-up.

**How might these results change the focus of research or clinical practice?**

- Our results support the importance of considering psychosocial health in the development and implementation of translational diabetes prevention programs in order to improve effectiveness among participants.

## Introduction

Type 2 diabetes mellitus (T2DM) is a chronic disease of global public health concern that is strongly associated with excess body weight and associated modifiable lifestyle factors, that is, poor diet and physical inactivity.^1^ In order to slow disease incidence, over the past few decades several randomized controlled trials tested the efficacy of lifestyle interventions for preventing T2DM among individuals at high risk for developing T2DM, which overall yielded strong positive findings.^2–6^ The largest and most diverse of these studies, the US Diabetes Prevention Program (DPP), found that an intensive lifestyle intervention targeting modest weight loss and increased physical activity reduced the incidence of type 2 diabetes by 58% over an average follow-up of 2.8 years as compared with placebo.^3^

The success of these trials has prompted efforts to translate the DPP framework to more ‘real-world’ settings. However, recent systematic reviews and meta-analyses of such translational trials found that there was considerable interstudy variation in program effectiveness for achieving weight loss and/or diabetes risk reduction.^7 8^ Although this may be explained by a number of factors, such as program adherence, intensity, or delivery, another emerging area of research that may be applicable is on the role of psychosocial factors in promoting or hindering behavior change in the setting of lifestyle interventions.

Self-efficacy (SE), or an individual’s confidence in their ability to perform a task, is one widely studied psychosocial construct in health behavior research.^9^ Using data from a substudy of participants in the original DPP, Delahanty *et al* 10﻿^*﻿*^ found that self-reported exercise SE at baseline was independently associated with higher levels of leisure physical activity at 1 year and at the end of the study (2–3 years after randomization). Greater exercise SE at baseline was also a significant predictor of achieving the 7% weight loss goal at the end of the study.^10 11^ In addition to baseline SE scores, 6-month improvements in low-fat dietary SE as a result of the intervention were associated with achieving 7% weight loss at the end of the study.^11^ This would suggest that individuals with higher SE at baseline or with greater improvements in SE as a result of a diabetes prevention program may be more responsive to lifestyle interventions, although this has not been adequately examined yet in the context of translational diabetes prevention research.

Another important gap in the literature is that very few, if any, studies of this nature have been conducted in low-income to middle-income countries to determine if these findings from high-income countries are applicable to other populations. The Diabetes Community Lifestyle Improvement Program (D-CLIP) was a randomized controlled research trial that tested the effectiveness of a translational diabetes prevention program with metformin when needed for preventing diabetes in overweight or obese Asian Indian adults with pre-diabetes, defined by impaired fasting glucose (IFG) and/or impaired glucose tolerance (IGT). Prior analyses have shown that D-CLIP resulted in a 32% reduction in diabetes incidence in the treatment group over a 3-year follow-up compared with control.^12^ We have also reported on baseline, cross-sectional data from this cohort, and found that SE levels were associated with physical activity levels and fruit and vegetable intake, and inversely associated with body mass index (BMI) and waist circumference (WC).^13^ In this study we investigated longitudinal changes in self-reported dietary and exercise SE from baseline to intervention completion (4 months), as well as annually until the end of the study (year 3). We also examined whether SE at baseline or improvements after the 4-month intervention were associated with reduced incidence of T2DM (the primary outcome) or greater success in achieving improvements in several secondary outcomes including weight, WC, exercise levels, and total energy intake.

## Research design and methods

### Parent study

The D-CLIP trial (ClinicalTrials.gov NCT01283308) was a translational, randomized controlled research study in Chennai, India described in detail previously.^14^ All participants provided written informed consent prior to screening, baseline testing, and study enrollment. The sample included men and women aged 20–65 years old who were overweight or obese, defined according to the WHO’s Asian-specific cut points for BMI (>23 kg/m^2^) or WC (≥90 cm for men or ≥80 cm for women), and diagnosed with pre-diabetes, defined according to the American Diabetes Association (ADA) criteria for IFG (fasting plasma glucose 5.6–6.9 mmol/L), IGT (2-hour, postload glucose of 7.8–11.0 mmol/L), or both.^15 16^ After enrollment, participants were randomized to lifestyle treatment or control. For this secondary analysis, we excluded non-compliant individuals who were lost to follow-up after the baseline visit (n=28), resulting in a final sample of 269 treatment and 281 control participants.

### Study interventions

The D-CLIP lifestyle treatment consisted of group-based, culturally appropriate lifestyle classes adapted from the original DPP.^14^ Briefly, it included 16 weekly core intervention classes on active lifestyle changes (months 0–4), followed by 8 maintenance classes (months 5–6). The two study goals were ≥7% weight loss and ≥150 min weekly of moderate-intensity exercise. After 4 months of the core intervention, lifestyle participants were prescribed metformin (500 mg twice daily) if they were at high risk of conversion to diabetes, defined as having both IFG and IGT or IFG and hemoglobin A1c ≥5.7% (39 mmol/mol). After 6 months when all classes were complete, contact with study staff was minimal, except at follow-up study visits. Control participants received the study site’s standard of care for pre-diabetes, which included a single day of one-on-one visits with a physician, a dietitian, and a fitness trainer, and one group class on diabetes prevention, but no additional contact except at study visits. No control arm participants received metformin because it was not part of standard of care at the study site.

### Measurements

Study visits took place at baseline, postcore intervention (4 months), postmaintenance intervention (6 months), 12 months, and every 6 months until study closeout (3–4 years after randomization) or diabetes diagnosis. For this analysis, we only used data collected up to year 3, although a small number of participants (n=81) were followed for another 6–12 months after this. Sociodemographic information was assessed at baseline by self-reported questionnaires. Pre-diabetes category and diabetes incidence were assessed by semiannual fasting blood draws at baseline and every 6 months and/or by oral glucose tolerance tests (OGTTs) at baseline and annually using the ADA criteria.^15^ Anthropometrics, including weight, height, and WC, were assessed by physical exams at all study visits, and weight and height were used to calculate the BMI. Physical activity levels were estimated in terms of weekly minutes of exercise, which were calculated using survey questions asking individuals how many days per week they exercise and how long each exercise session lasts on average. Total energy intake was assessed at annual visits by a food frequency questionnaire developed for South Indian populations.^17^

### Psychosocial assessments

SE was assessed by self-reported surveys at baseline, 4 months, and at annual visits. Exercise SE was measured using an instrument developed by Sallis *et al* 18, which asked about an individual’s perception that he/she has the ability to exercise in 12 different situations using a 5-point Likert-type scale. The instrument provided scores for exercise SE on two scales: ‘sticking to it’ (adhering to an exercise regimen regardless of mood and situation) and ‘making time’ (prioritizing exercise over other time demands). These two subscores were summed to yield a total exercise SE score. Dietary SE was measured using the Weight Efficacy Lifestyle (WEL) questionnaire.^19^ This instrument assesses an individual’s confidence in his/her ability to avoid overeating using a 10-point Likert-type scale. The WEL provides a total score as well as scores on five subscales: negative emotions, availability, social pressure, physical discomfort, and positive activities.

### Statistical analysis

All statistical analyses were performed in SAS V.9.4 (SAS Institute Inc., Cary, NC, USA). Baseline total and component scores for dietary and exercise SE, as well as other characteristics of the sample, were summarized as mean±SD and counts (percentages). Mixed-effects regression was used to calculate changes in SE total and component scores over time by treatment group. Each model included participants as a random effect, and time, treatment group, and a time × treatment group interaction as fixed effects. Other covariates including sex and baseline age, BMI, and pre-diabetes type were included as fixed effects. Time was treated as a discrete ordinal variable because there was evidence of a non-linear pattern of change over time for the psychosocial variables. Within-group differences were assessed by comparing least square means (LS-means) at each time point (4, 12, 24, and 36 months) to baseline, and between-group differences were assessed by comparing LS-means between groups at each time point. LS-mean differences and 95% CI are reported, as well as p values adjusted for multiple comparisons by the Tukey-Kramer method.

For the subsequent analyses, the analytical sample was limited to treatment participants to test intervention-specific associations. First, Cox proportional hazard models were used to assess whether SE at baseline or SE change from baseline to intervention completion (at 4 months) were associated with time to T2DM, adjusting for sex and baseline age, BMI, and pre-diabetes type. Both exercise and dietary SE variables were entered into the model as pairs consisting of baseline and 4-month change values, which were calculated as SE score at 4 months minus SE score at baseline. Date of last follow-up was marked by either diagnosis of T2DM or censorship due to study completion (3 years) or dropout.

Lastly, associations of SE with secondary outcomes, that is, change in weight, WC, weekly exercise, and total energy intake, at 4, 12, 24, and 36 months were evaluated using linear regression. Similar to above, SE variables were entered into the model as baseline values and 4-month change values. Models were adjusted for sex, and baseline age, BMI, and pre-diabetes type. For the models with 12-month, 24-month, and 36-month changes as the outcome, the analytical sample was limited to only participants who had follow-up data at that time point, and a covariate for exercise or dietary SE score at that time point was included in the model. Beta coefficients are reported as standardized coefficients and standard errors, and statistical significance was set at p<0.05.

## Results

**Figure 1:**
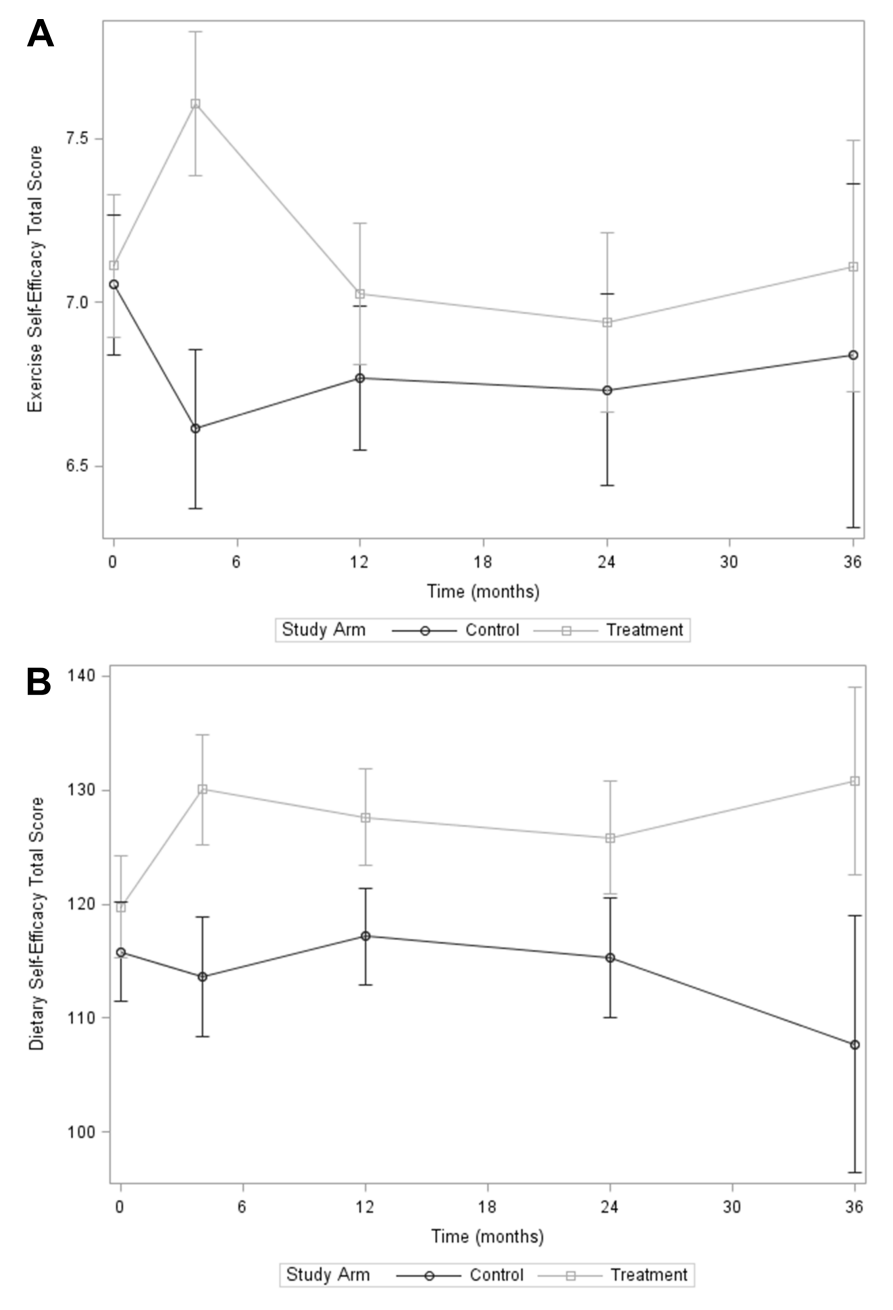
Total scores for psychosocial variables over 3-year follow-up by treatment group: (A) exercise self-efficacy and (B) dietary self-efficacy. Markers represent the least square mean for each group and time, adjusted for sex and baseline age, body mass index, and pre-diabetes type. Error bars represent 95% CIs. Circles: control group; squares: treatment group.

Baseline health and psychosocial characteristics of the sample are summarized in table 1. There were no significant differences between groups for exercise or dietary SE total or component scores at baseline. In mixed-effects models, exercise and dietary SE total scores increased significantly from baseline to 4 months within the treatment group (LS-mean difference (95% CI), 4 months vs baseline: 0.49 (0.20 to 0.79) for exercise SE, adjusted p (adj-p)=0.04; 10.3 (4.0 to 16.6) for dietary SE, adj-p=0.04) but not in the control group (figure 1). There was also a significant between-group difference in exercise and dietary SE at 4 months (LS-mean difference (95% CI), treatment vs control: 0.99 (0.67 to 1.32) for exercise SE, adj-p<0.001; 16.4 (9.4 to 23.5) for dietary SE, adj-p<0.001). However, over long-term follow-up (≥12 months), within-group differences at each time point compared with baseline were no longer significant for either exercise or dietary SE, and between-group differences were no longer significant for exercise SE. For dietary SE, the treatment group sustained a higher total score compared with the control group at 12 and 36 months (LS-mean difference (95% CI), treatment vs control: 10.4 (4.5 to 16.4) for 12 months, adj-p=0.02; 23.1 (9.2 to 37.1) for 36 months, adj-p=0.04) (figure 1). Similar trends were seen in the exercise and dietary SE component scores (online supplementary figures S1 and S2).

10.1136/bmjdrc-2018-000561.supp1 Supplementary data

**Table 1:** Demographic, health, and psychosocial characteristics of the analytical sample at baseline by treatment group (N=550)

	Control (n=281)	Treatment (n=269)
Female, n (%)	110 (39.2)	96 (35.7)
Male, n (**%**)	171 (60.9)	173 (64.3)
Age (years), mean±SD	44.3±9.4	44.9±8.8
Anthropometrics, mean±SD		
BMI (kg/m^2^)	27.9±3.7	28.0±3.7
Male WC (cm)	90.1±8.3	89.9±9.1
Female WC (cm)	97.7±7.7	97.8±8.2
Pre-diabetes type, n (%)		
IGT	81 (28.8)	81 (30.1)
IFG	81 (28.8)	85 (31.6)
IGT+IFG	119 (42.4)	103 (38.3)
Dietary self-efficacy, mean±SD		
Positive activities score	23.6±9.5	23.8±8.8
Availability score	21.0±9.6	22.0±9.4
Physical discomfort score	24.4±8.9	25.0±8.8
Negative emotions score	23.8±9.2	24.7±8.9
Social pressure score	22.1±8.9	23.3±8.5
Total score	114.9±38.9	118.7±37.3
Exercise self-efficacy, mean±SD		
Sticking to it	3.4±1.0	3.4±1.0
Making time	3.7±1.0	3.8±1.0
Total scores	7.1±1.8	7.2±1.8
Physical activity, mean±SD		
Exercise, min/week	77.4±103.3	84.2±113.9
Dietary intake, mean±SD		
Total daily energy intake	3001±838	2950±891

BMI, body mass index; IFG, impaired fasting glucose; IGT, impaired glucose tolerance; WC, waist circumference.

In our analyses of treatment participants only, there was no association of baseline or initial 4-month change in dietary and exercise SE with T2DM incidence over the 3-year follow-up in Cox proportional hazard models (online supplementary table S1). Table 2 reports the standardized beta coefficients, standard errors, and p values for the associations of baseline and initial 4-month change in SE scores with secondary outcomes among treatment participants at 4, 12, 24, and 36 months of follow-up. For short-term (4 months) outcomes, baseline scores for exercise SE were significantly associated with decreased weight and WC, and increased exercise at 4 months among treatment participants in linear regression adjusted for sex, and baseline age, BMI, and pre-diabetes type (all p<0.05). Initial 4-month change in exercise SE was also associated with increased exercise at 4 months (p<0.001). For secondary outcomes at 12-month, 24-month or 36-month follow-up, neither baseline nor initial 4-month change in dietary and exercise SE predicted weight change at any time point. However, the covariates for dietary and exercise SE at 12 months were associated with decreased weight at 12 months; the same was true for dietary SE and weight at 24 months (p<0.05). For the other outcomes, initial 4-month change in exercise SE significantly predicted decreased WC at 24 months, and increased exercise at 12 and 24 months among treatment participants (p<0.05). None of the variables for dietary nor exercise SE were associated with changes in energy intake at any time point (table 2).

**Table 2:** Estimates from linear regression of dietary and exercise SE total scores with change in secondary health outcomes at 4, 12, 24, and 36 months among treatment participants*†

SE variable	Weight (kg)		WC (cm)		Exercise (min/week)		Energy intake (kcal)	
	β (standard error)	P values	β (standard error)	P values	β (standard error)	P values	β (standard error)	P values
	**4 months (n=238)**		**4 months (n=237)**		**4 months (n=241)**		**4 months (n=195)**	
Dietary SE								
Baseline	−0.02 (0.01)	0.81	−0.03 (0.01)	0.73	0.02 (0.27)	0.77	−0.02 (2.19)	0.82
4-month Δ	−0.02 (0.01)	0.84	0.02 (0.01)	0.80	0.05 (0.27)	0.50	−0.12 (2.21)	0.20
Exercise SE								
Baseline	**−0.22 (0.14)**	**0.02**	**−0.19 (0.24)**	**0.04**	**0.22 (6.0)**	**0.01**	−0.01 (47.8)	0.91
4-month Δ	−0.15 (0.12)	0.10	−0.15 (0.20)	0.11	**0.39 (5.2)**	**<0.01**	0.08 (41.4)	0.43
	**12 months (n=235)**		**12 months (n=235)**		**12 months (n=233)**		**12 months (n=205)**	
Dietary SE								
Baseline	−0.02 (0.01)	0.76	−0.14 (0.01)	0.11	0.06 (0.35)	0.49	−0.07 (2.33)	0.49
4-month Δ	−0.1 (0.01)	0.32	−0.07 (0.01)	0.40	−0.06 (0.32)	0.49	0.03 (2.05)	0.75
12 months	**−0.13 (0.01)**	**0.01**	−0.07 (0.01)	0.38	−0.02 (0.32)	0.79	0.15 (2.11)	0.08
Exercise SE								
Baseline	−0.03 (0.19)	0.20	−0.19 (0.29)	0.07	0.01 (8.47)	0.95	−0.02 (53.66)	0.88
4-month Δ	0.001 (0.15)	0.19	−0.15 (0.23)	0.14	**0.24 (6.66)**	**0.02**	−0.06 (41.84)	0.57
12 months	**−0.22 (0.15)**	**<0.01**	−0.08 (0.23)	0.28	0.06 (6.61)	0.48	−0.06 (43.34)	0.49
	**24 months (n=207)**		**24 months (n=207)**		**24 months (n=199)**		**24 months (n=186)**	
Dietary SE								
Baseline	0.13 (0.01)	0.17	0 (0.01)	0.99	−0.08 (0.31)	0.42	0.18 (2.41)	0.07
4-month Δ	0.117 (0.01)	0.19	0.06 (0.01)	0.47	−0.06 (0.28)	0.49	0.12 (2.28)	0.22
24 months	**−0.18 (0.01)**	**0.03**	−0.1 (0.01)	0.24	0.03 (0.29)	0.71	0.06 (2.22)	0.52
Exercise SE								
Baseline	−0.11 (0.24)	0.34	−0.2 (0.33)	0.06	0.028 (6.98)	0.80	0.2 (53.12)	0.09
4-month Δ	−0.13 (0.2)	0.25	**−0.29 (0.27)**	**0.01**	**0.26 (5.67)**	**0.02**	0.14 (44.21)	0.21
24 months	−0.01 (0.19)	0.94	0.02 (0.25)	0.79	0.15 (5.26)	0.06	−0.12 (41.15)	0.17
	**36 months (n=100)**		**36 months (n=100)**		**36 months (n=99)**		**36 months (n=89)**	
Dietary SE								
Baseline	0.07 (0.02)	0.66	0.06 (0.02)	0.69	−0.14 (0.52)	0.34	0.13 (3.51)	0.37
4-month Δ	−0.04 (0.01)	0.74	−0.05 (0.02)	0.72	−0.12 (0.43)	0.38	−0.119 (2.96)	0.37
36 months	−0.11 (0.01)	0.40	−0.07 (0.02)	0.60	0.08 (0.46)	0.56	−0.07 (3.1)	0.56
Exercise SE								
Baseline	−0.15 (0.38)	0.44	−0.12 (0.48)	0.52	0.22 (13.44)	0.26	0.31 (90.42)	0.11
4-month Δ	−0.1 (0.33)	0.61	−0.04 (0.41)	0.83	0.37 (11.38)	0.07	0.28 (78.02)	0.17
36 months	−0.06 (0.28)	0.65	−0.04 (0.35)	0.74	−0.08 (9.54)	0.51	0 (66.83)	0.98

*Models were adjusted for sex, and baseline age, body mass index, and pre-diabetes type. Estimates are standardized beta coefficients.

†Change values for each secondary outcome and for 4-month change in SE scores were calculated as follow-up baseline value.

‡Bold values indicate a statistically siganificant association at p<0.05.

SE, self-efficacy; WC, waist circumference.

## Discussion

This study expanded on previous findings from the D-CLIP trial by examining the role of psychosocial variables as correlates of health outcomes among Asian Indian adults with pre-diabetes in a stepwise diabetes prevention program (culturally adapted, group-based, DPP-like program with metformin if needed). Consistent with earlier translational research,^20–26^ the D-CLIP program resulted in higher scores for exercise and dietary SE for the treatment group compared with the control group at completion of the core intervention. However, these increases in SE within the treatment group were not maintained over long-term follow-up, and returned near baseline levels after 4 months. Nonetheless, there remained a significant difference in dietary SE between the treatment and control groups at 12 and 36 months. However, there were no differences between groups for exercise SE at 12 months or after. These findings indicate that certain SE beliefs may require continual reinforcement to sustain in the long term. During the lifestyle intervention, participants had weekly group-based exercise classes. It is possible that active participation in these classes overcame some of the barriers to exercise and promoted increased exercise SE, which was not sustained once these classes stopped.^27^ Other studies have also reported inconsistent findings on long-term changes in SE after a translational DPP-style intervention. While some did find differences at 12 months for certain measures of SE,^28^ other studies found no long-term differences,^22 23^ and no studies to our knowledge have reported on longer term (≥2 years) follow-up of SE scores. Additional qualitative research may be warranted to elucidate the individual and/or program-related factors that explain why some psychosocial beliefs are sustained over the long term while others are not.

In our analysis of the relationship of SE and diabetes-related health outcomes among treatment participants, we did not observe significant associations of SE at baseline, nor 4-month improvement in SE as a result of the intervention, with incidence of T2DM over follow-up. This would mean that the relative success among treatment participants in achieving this outcome, that is, prevention of T2DM, was more strongly related to factors other than SE, such as pre-diabetes type, age, and sex, as reported previously.

For secondary outcomes, exercise SE at baseline was a significant predictor of improved weight and WC at completion of the D-CLIP core intervention at 4 months, supporting that individuals with higher exercise SE going into the intervention experienced greater success with improving obesity-related outcomes. We also found that improved weight at later time points, that is, 12 and 24 months, was significantly related to dietary and/or exercise SE at these time points. These findings are similar to that of other studies showing that SE is a correlate or mediator of weight and WC changes in a DPP-style intervention.^22–24 29^ Exercise SE at baseline also predicted increased exercise levels at 4 months, while initial 4-month change in exercise SE predicted increased exercise levels at 12 and 24 months. This suggests that greater SE in part explains interindividual differences in improvements in physical activity among participants who received the D-CLIP intervention. This also mirrors findings from other studies, such as the Special Diabetes Program for Indian Diabetes Prevention, which found that participants with higher SE were more likely to be categorized in the ‘Action-Maintenance’ stage (defined by the transtheoretical model of behavioral change), and exhibited higher physical activity and healthier diets compared with the ‘Contemplation’ and ‘Preparation’ stages.^30 31^ This alignment of improved exercise SE with improved physical activity is logical, and suggests that careful attention should be paid to the type of SE being measured in future studies depending on the health outcomes of interest, as these differences may explain discrepancies when comparing study findings. For example, Gillison *et al* 28 found significant associations of SE with dietary change, motivation and social support in a group-based translational lifestyle intervention, but not change in physical activity levels, which may be because SE was measured in this study in relation to dietary, but not physical activity behaviors.

In contrast to the aforementioned studies, we found no associations of exercise or dietary SE with change in energy intake at any time point. It is possible that, similar to T2DM incidence, success in reducing energy intake over follow-up may have been more strongly related to other factors. Limitations in the instruments used to measure energy intake and SE may also have influenced this finding. First, the food frequency questionnaire used to measure dietary intake relied on self-reported frequencies and portion sizes, which can be subject to recall bias and social desirability bias,^32 33^ especially in participants with higher BMI,^17^ thereby potentially limiting our ability to accurately estimate energy intake. However, it is not likely that this significantly impacted the results as the measurement tools used were validated and were able to provide relative measurements of these variables. Alternatively, it is possible that the instruments used to measure SE did not provide sensitive estimates of these variables, as neither the exercise nor dietary SE questionnaires have been validated specifically for the Indian population. Future studies aiming to develop and validate an instrument for measuring health-related SE in this population may be warranted.

Other limitations of this analysis included the use of brief questionnaires for assessing psychosocial, dietary, and physical activity variables, which may have been too simple to provide accurate measurements. The self-report surveys were also subject to the biases of self-reported data as previously explained. Another limitation was that participants were no longer followed after being diagnosed with T2DM; thus, we had no data on their clinical course after diagnosis and this limited our sample size for analyses of outcomes over a long-term follow-up. Finally, we only assessed certain psychosocial constructs in this study to reduce respondent burden, and thus did not capture the intervention’s effects on other psychosocial predictors of behavioral change. Future studies may benefit from conducting a full battery of cognitive tests, including other variables such as planning or knowledge variables, which in some studies were a more important predictor of health behavior change than SE.^22 34^

The D-CLIP trial has several strengths. It used a randomized controlled trial design, and the sample was balanced after randomization. The trial had a longer duration of follow-up than most translational trials of diabetes prevention program (up to 4 years for some participants), allowing us to understand the longer term impact of a translational diabetes prevention program over time and after the core and maintenance intervention was complete. This study was one of the first to examine the role of psychosocial factors in diabetes prevention and related health outcomes among Asian Indian adults, a population at higher risk for T2DM at younger ages and lower BMIs.^35^ The sample population also included all forms of pre-diabetes, adding a novel perspective to our understanding of the mechanisms and impact of a community-based diabetes prevention program in this region. The translational nature of the intervention was also a strength in that the D-CLIP classes were designed to be delivered in a way that is lower in cost and less resource-intensive than individualized programs.

## Conclusions

This study provides additional insights into the potential role of psychosocial factors, in particular SE, in predicting success in achieving primary and secondary outcomes within a community-based, translational diabetes prevention program. Although SE did not impact the risk of developing type 2 diabetes, baseline SE and/or initial improvements in SE as a result of the intervention predicted improved weight loss, reductions in WC, and exercise. Future studies are needed to better understand the specific mechanisms by which psychosocial factors mediate the associations of DPP-style interventions with improved health outcomes as this may also help to improve the effectiveness of interventions.
