# Supplementary material for: Self-efficacy and diabetes prevention in overweight South Asians with pre-diabetes
Source: BMJ Open Diabetes Res Care. 2018 Oct 15;6(1):e000561. doi: 10.1136/bmjdrc-2018-000561 (PMC6203034; doi:10.1136/bmjdrc-2018-000561)
Supplement: Supplementary data [file bmjdrc-2018-000561supp001.docx]

**SUPPLEMENTARY TABLES & FIGURES**


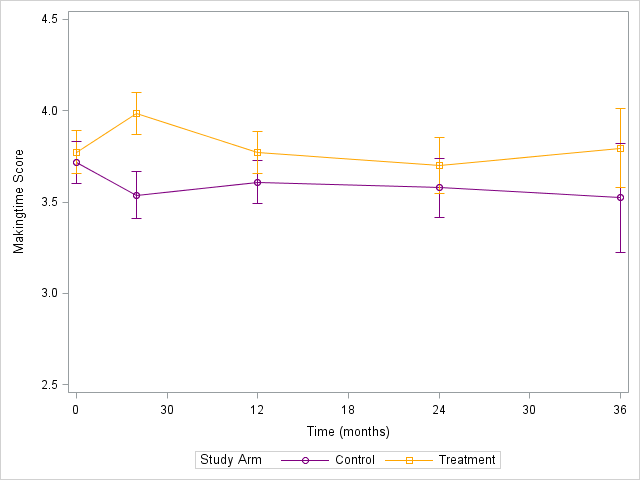

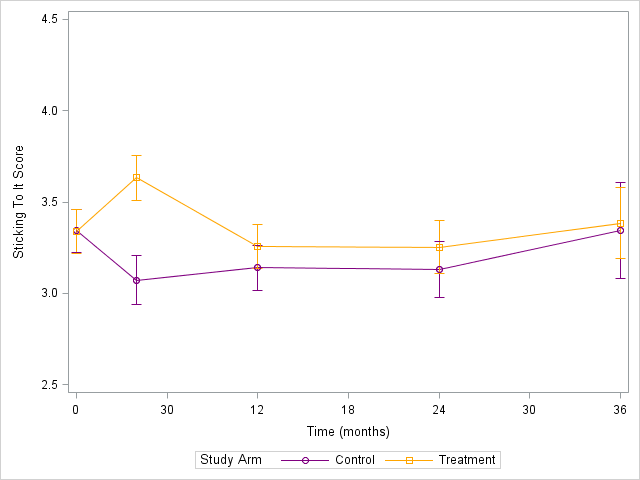


(a) (b)

**Figure S1: S**cores for exercise self-efficacy sub-scales over 36-months of follow-up by treatment group. (a) “Making Time” sub-scale; (b) “Sticking To It” sub-scale. Markers represent the LS-mean for each group and time, adjusted for sex and baseline age, BMI, and prediabetes type. Error bars represent 95% confidence intervals.


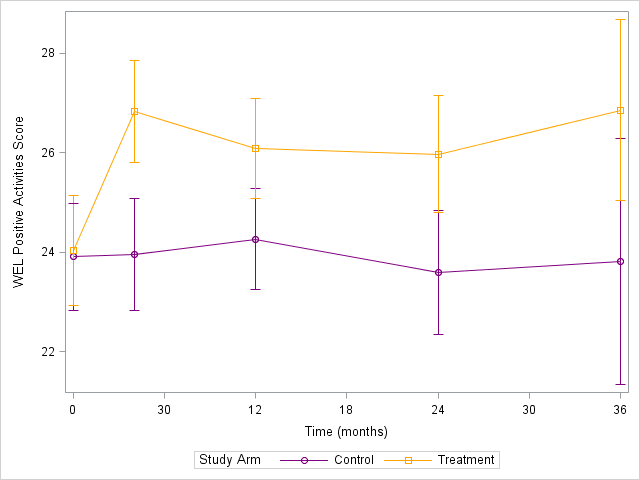

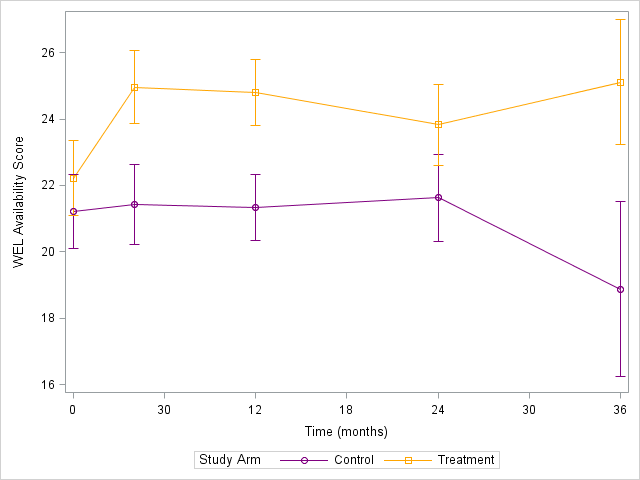


(a) (b)


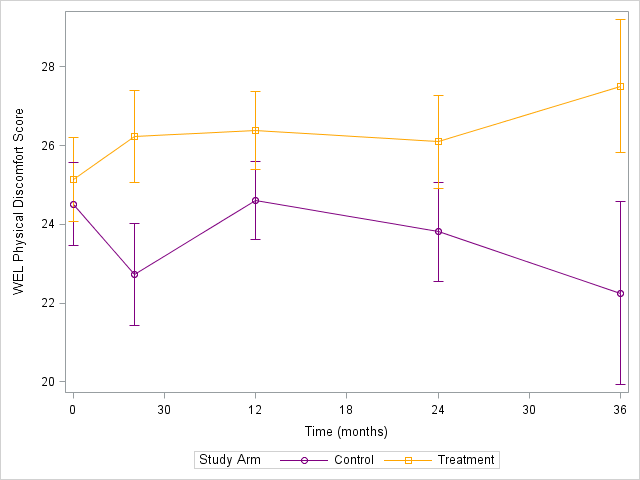

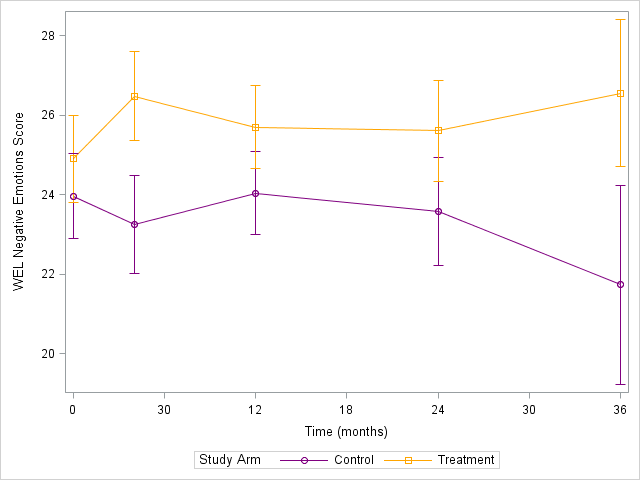


(c) (d)


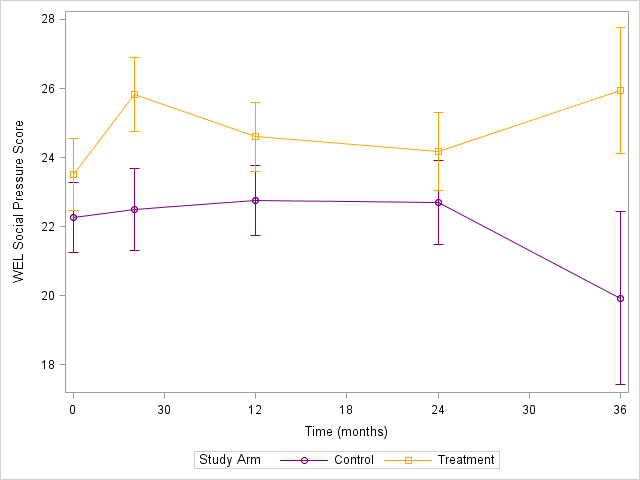


(e)

**Figure S2: S**cores for WEL eating self-efficacy sub-scales over 36-months of follow-up by treatment group. (a) “Positive Activities” sub-scale; (b) “Availability” sub-scale; (c) “Physical Discomfort” sub-scale; (d) “Negative Emotions” sub-scale; (e) “Social Pressure” sub-scale. Markers represent the LS-mean for each group and time, adjusted for sex and baseline age, BMI, and prediabetes type. Error bars represent 95% confidence intervals.

| Table S1: Estimates from adjusted cox proportional hazard model relating psychosocial variables to time to T2DM diagnosis among treatment group participants (n=269) | | | |
| --- | --- | --- | --- |
| Parameter | **Β (SE)** | **P-value** | **HR (95% CI)** |
| Dietary SE: Baseline | -0.003 (0.004) | 0.43 | 1.00 (0.99, 1.01) |
| Dietary SE: 4-month change | -0.002 (0.004) | 0.61 | 1.00 (0.99, 1.01) |
| Exercise SE: Baseline | -0.14 (0.1) | 0.15 | 0.87 (0.72, 1.05) |
| Exercise SE: 4-month change | -0.1 (0.08) | 0.22 | 0.90 (0.77, 1.06) |
| Treatment Group | -0.17 (0.28) | 0.53 | 0.84 (0.49, 1.44) |
| Sex | -0.01 (0.01) | 0.53 | 0.99 (0.96, 1.02) |
| Baseline Age | 0.03 (0.03) | 0.34 | 1.03 (0.97, 1.11) |
| Baseline BMI | -0.73 (0.3) | 0.02 | 0.48 (0.27, 0.88) |
| iIGT (Vs. both) | -0.84 (0.32) | 0.01 | 0.43 (0.23, 0.81) |
| iIFG (Vs. both) | -0.003 (0.004) | 0.43 | 1.00 (0.99, 1.01) |
